# Correlation of Zinc with Oxidative Stress Biomarkers

**DOI:** 10.3390/ijerph120303060

**Published:** 2015-03-12

**Authors:** María Morales-Suárez-Varela, Agustín Llopis-González, Verónica González-Albert, Raúl López-Izquierdo, Isabel González-Manzano, Javier Cháves, Vicente Huerta-Biosca, Juan C. Martin-Escudero

**Affiliations:** 1 Unit of Public Health, Hygiene and Environmental Health, Department of Preventive Medicine and Public Health, Food Science, Toxicology and Legal Medicine, University of Valencia, 46100 Valencia, Spain; E-Mails: agustin.llopis@uv.es (A.L.-G.); vicente.huerta@uv.es (V.H.-B.); 2 CIBER Epidemiología y Salud Pública (CIBERESP), Instituto de Salud Carlos III, 28029 Madrid, Spain; 3 Center for Advanced Research in Public Health (CSISP-FISABIO), 46010 Valencia, Spain; 4 Genotyping and Genetic Diagnosis Unit; Hospital Clinic Research Foundation and INCLIVA, University of Valencia, 46010 Valencia, Spain; E-Mails: veronica.gonzalez@uv.es (V.G.-A.); felipe.chaves@uv.es (J.C.); 5 Internal Medicine Department, Rio Hortega University Hospital, 47012 Valladolid, Spain; E-Mails: rulo636@yahoo.es (R.L.-I.); isabelgonzlez@gmail.com (I.G.-M.); juancarlos.martinescudero@gmail.com (J.C.M.-E.); 6 CIBER de Diabetes y Enfermedades Metabólicas Asociados (CIBERDEM), Instituto de Salud Carlos III, 28029 Madrid, Spain

**Keywords:** zinc, oxidative stress, hypertension, cellular aging

## Abstract

Hypertension and smoking are related with oxidative stress (OS), which in turn reports on cellular aging. Zinc is an essential element involved in an individual’s physiology. The aim of this study was to evaluate the relation of zinc levels in serum and urine with OS and cellular aging and its effect on the development of hypertension. In a Spanish sample with 1500 individuals, subjects aged 20–59 years were selected, whose zinc intake levels fell within the recommended limits. These individuals were classified according to their smoking habits and hypertensive condition. A positive correlation was found (Pearson’s C = 0.639; *p* = 0.01) between Zn serum/urine quotient and oxidized glutathione levels (GSSG). Finally, risk of hypertension significantly increased when the GSSG levels exceeded the 75 percentile; OR = 2.80 (95%CI = 1.09–7.18) and AOR = 3.06 (95%CI = 0.96–9.71). Low zinc levels in serum were related with OS and cellular aging and were, in turn, to be a risk factor for hypertension.

## 1. Introduction

Hypertension is prevalent in the community and is a risk factor for several cardiovascular diseases, including sudden death, stroke, coronary heart disease, heart failure, atrial fibrillation and peripheral artery disease [1]. High blood pressure came in fourth place as a risk factor associated with mortality in 1990 [2], while it was the first risk factor associated with mortality in 2010. The mortality rate in Spain in 2008 among adults aged 30–70 years due to cardiovascular diseases and diabetes was 68 deaths for every 100,000 inhabitants [3], which makes such disorders the second cause of death behind cancer. It has also been shown that hypertension accelerates physiological cellular aging, causing left atrial dilation [4].

Regulation in the human organism of OS levels is a complex matter. Of the factors that can increase OS, we find alteration of some essential metals [5], smoking and presence of hypertension (although increased OS can also cause hypertension). However, the importance attached to each one has not yet been well-established. Trace elements, which include zinc, must be present in the body in suitable quantities.

Zinc is an essential nutrient for humans because it is involved in the functionality of many metalloenzymes, and is also vital given its important antioxidant properties [6]. Zinc participates in major body functions, like protein synthesis, DNA synthesis and cell growth. Zinc plays a key role in the immunological system [6]. It is absorbed in the small intestine and is transported in blood along with albumin [7].

Although the harmful effects of cigarette smoke on cardiovascular morbidity and mortality have been well-established, the appearance and/or temporary progression of processes and manifestations of physiological and pathological disorders induced by smoking are not well-known [8].

Tobacco leaves naturally accumulate and concentrate relatively high levels of Cu, Cd, Pb and Ni if compared with medicinal and edible plants, which contain much lower levels of these metals. Therefore, smoking tobacco is a major source of exposure to the above metals for smokers. The total quantity of carcinogenic substances in tobacco smoke varies from 1 to 3 µg per cigarette [9]. It has been identified that smoking modifies the levels of zinc in the body [10]. Many studies [11] have confirmed the relationship between smoking and increased risk of cardiovascular disease.

A free radical is defined as any molecular species that is capable of existing independently and contains one unpaired electron in an atomic orbital [12]. Under normal physiological conditions, a balance is maintained between oxidants and endogenous antioxidants. When imbalance occurs, created by either excessive oxidant generation or reduced antioxidants, the abnormal oxidant system then enters a state known as OS [13]. The balance between reactive oxygen species (ROS) production and antioxidant mechanisms is altered in aging, and also in several neurodegenerative diseases like Alzheimer’s, Parkinson’s or schizophrenia [14].

The most widely used marker to measure damage caused to DNA by OS in human biomonitoring studies is presence of 8-hydroxydeoxyguanosine (8-OHdG) in the DNA of leukocytes and excretion of 8-OHdG in urine [15]. Presence of 8-OHdG in urine is considered a major biomarker of generalized cellular OS and a product of DNA repair [13].

Oxidative stress can intensify lipid peroxidation. Malondialdehyde (MDA), one of the aldehydes produced during lipid peroxidation, is known to be a final product of free radical reactions. High MDA levels are found in many illnesses, including cardiovascular disease and cancer [16]. Glutathione (GSH, Glutamyl-L-cysteinylglycine) is the most abundant antioxidant at the intracellular and multifunctional levels, and is present in all living organisms. Glutathione comes in two forms: GSH (reduced) and GSSG (oxidized) [17].

Although zinc has antioxidant properties [6], previous studies [18] have found a correlation between presence and increased levels of OS markers. Hence how OS is associated with this trace element has not yet been defined. This study aims to evaluate the relation of Zn levels in blood serum and urine with OS and cellular aging and its effect on the development of hypertension in smoking and non-smoking Spanish populations.

## 2. Materials and Methods

### 2.1. Study Population

To carry out this study, we used a database with 1500 individuals from the Spanish city of Valladolid. All the participants gave their written consent to participate in this study before 24-hour urine samples were taken.

**Figure 1:**
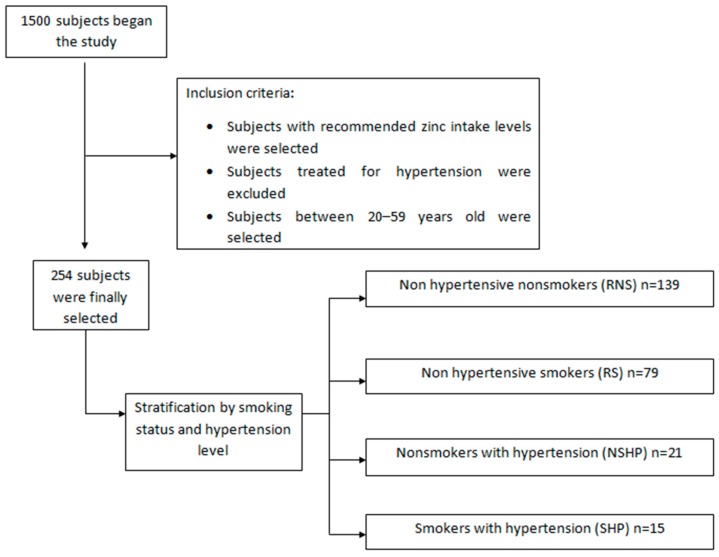
Flowchart of the study population.

To the study population of 1500 individuals, some inclusion criteria were applied. First, those subjects who did not present the recommended daily intake (RDI) Zn levels [19] (9.5–12 mg/day for men, 7–12 mg/day for women) were excluded. Next, any subjects diagnosed with hypertension, and who were on treatment, were ruled out as it was considered that this could alter the result of the study. Finally, the subjects in the 20–59 year-old age group were selected to avoid the effect that age might have on variation in zinc levels. Thus the final study population comprised 254 individuals (Figure 1) and a number of confounding factors were controlled.

Afterward the study population was stratified by their smoking habits and level of hypertension. A reference value was set to distinguish between smoking and non-smoking individuals. This limit of separation between smokers and non-smokers was set at 10 ng/mL of cotinine [20] (a metabolite of tobacco) in blood serum. Systolic and diastolic blood pressure values were taken in accordance with the criteria set out in the literature [21] for gender and age, which indicate levels equal to or higher than 140 mm Hg for systolic pressure, and of 90 mm Hg for diastolic pressure, as being indicative of hypertension. This allowed us to classify individuals into two categories: normotensive and hypertensive.

The application of these inclusion criteria, in combination with blood pressure levels and smoking status, gave four groups: non-smokers without hypertension (RNS); smokers without hypertension (RS); non-smokers with hypertension (NSHP); smokers with hypertension (SHP).

### 2.2. Determining Zinc in Biological Samples

The concentration of zinc and other metals was measured by the inductively couple plasma mass spectrometry technique (ICP-MS) in an Agilent 7500CE ICP-MS. The limits of detection (LOD) of zinc in blood plasma went from 4.22 to 17.34 µmol/L, and the LOD in urine was <0.08 µmol/g creatinine. The reference values determined for zinc in blood plasma went from 10.70 to 18.40 µmol/L.

### 2.3. Measuring Markers of Oxidative Stress

GSH, GSSG, MDA, and 8-OHdG were determined from urine samples. GSSG and GSH were analyzed by high-performance liquid chromatography (HPLC). MDA was analyzed by HPLC and by the spectrophotometric quantification of MDA-thiobarbituric acid (TBA) at 532 nm. The quantity of 8-OHdG in urine was measured by HPLC with electrochemical detection (HPLC-CE). The data of the OS biomarkers were corrected by creatinine and were indicated in nanomoles divided by millimoles of creatinine (nmol/mmol creatinine). The quantity of creatinine in urine was measured by the modified kinetic method of Jaffé [22].

### 2.4. Study Procedure

This study evaluated the effect of smoking on levels of zinc in the blood serum and urine of the hypertensive/non-hypertensive and smokers/non-smokers groups. A database with 1500 subjects was used. Subjects were selected in the health area covered by the Río Hortega University Hospital (214,445 inhabitants) in the province of Valladolid (NW Spain). This study was a cross-sectional survey designed in two phases. In the first phase, a questionnaire was mailed to a random sample of 20% of the population aged between 15 and 82 years, for whom cardiovascular risk factors were collected. In the second phase, a new questionnaire was sent out, and a programmed interview and physical examination were conducted. A random subsample consisting of 1500 people was selected. It was self-weighted and stratified by age, gender, level of education, declared body mass index (BMI) and other characteristics. Apart from socio-demographic data, data were collected from the SF-36 Health Questionnaire, a tobacco use questionnaire that reflects the number of cigarettes smoked and duration of smoking habit. No differences were found between collaborating and noncollaborating subjects if compared to the other variables. Participants were representative of the general population. All the participants gave their informed consent and agreed to participate in the study.

### 2.5. Statistical Analysis

The population was stratified into four groups: RNS, RS, NSHP and SHP. The results of the quantitative determinations were expressed as the mean ± standard deviation (SD). Differences between groups were analyzed using a Student’s t-test and a one-way analysis of variance (ANOVA) with a 95% confidence level of significance. Pearson’s correlation test was applied to determine the relations among the variables. In order to verify the association among parameters (age, BMI, gender, vitamin B12 intake and the Zn serum/urine quotient), multiple logistic regression models were run by calculating the crude and adjusted odds ratios (OR) for gender, age, BMI, vitamin B12 intake and Zn serum/urine quotient. Values of *p* < 0.05 were considered statistically significant. The analyses were done with the SPSS v19 (IBM SPSS Statistics for Windows, version 19.0. Released 2011. IBM Corp.: Armonk, NY, USA) and Epi Info 7 (CDC, Atlanta, GA, USA, 2011) software.

## 3. Results

### 3.1. Patients’ Characteristics

Table 1 indicates the main characteristics of the study population. Table 2 offers the prevalence of the pathologies in the study population, which provides further knowledge of the population sample’s characteristics. Table 3 offers the zinc and OS markers values of the study population. The Zn levels in serum lowered in the groups of hypertensive subjects. The RNS obtained the highest value (13.39 μmol/L), and the SHP gave the lowest one (12.23 μmol/L). The Zn values in urine were higher in hypertensive subjects, with the highest value found in the NSHP (2.78 μmol/L) and the lowest one in the RNS (2.55 μmol/L). The Zn serum/urine quotient values were higher in the non-hypertensive patient groups; the highest value was observed for RNS (43.74) and the lowest one went to SHP (15.40).

**Table 1:** Characteristics of hypertensive and non-hypertensive subjects.

Parameters	RNS (n = 139)		RS (n = 79)		NSHP (n = 21)		SHP (n = 15)	
	Mean ± SD	95%CI	Mean ± SD	95%CI	Mean ± SD	95%CI	Mean ± SD	95%CI
Height (cm)	159.10 ± 32.05	155.51–162.69	164.61 ± 8.24	163.1–166.1	165.67 ± 10.68	161.31–170.09	169.40 ± 9.28	164.83–173.97
Weight (kg)	64.59 ± 17.59	62.63–66.57	66.80 ± 12.20	64.56–69.04	75.81 ± 16.35	69.07–82.53	78.53 ± 11.49	72.84–84.16
BMI (kg/m^2^)	23.59 ± 5.60	22.97–24.23	24.60 ± 3.72	23.92–25.28	27.50 ± 4.68	25.57–29.43	27.56 ± 4.82	25.24–29.96
SBP (mmHg)	114.69 ± 15.74	112.94–116.46	113.22 ± 17.39	110.01–116.39	143.49 ± 11.57	138.74–148.26	133.51 ± 13.78	126.71–140.29
DBP (mmHg)	73.81 ± 10.19	72.66–74.94	73.67 ± 11.54	71.59–75.81	91.16 ± 5.73	88.86–93.54	86.58 ± 9.69	81.83–91.37
PP (mmHg)	40.94 ± 10.22	39.76–42.04	39.60 ± 10.53	37.67–41.53	52.31 ± 12.76	47.05–57.55	47.27 ± 14.78	40.02–54.58
Waist (cm)	81.73 ± 12.20	80.33–83.07	81.50 ± 10.48	79.57–83.43	89.72 ± 12.24	84.69–94.71	91.43 ± 8.01	87.47–95.33
Hip (cm)	99.60 ± 6.11	98.92–101.28	101.51 ± 14.93	98.77–104.23	104.06 ± 10.17	99.91–108.29	104.79 ± 8.96	100.37–109.23
Waist/hip ratio	0.83 ± 0.09	0.79–0.81	0.81 ± 0.09	0.78–0.82	0.86 ± 0.09	0.86–0.94	0.88 ± 0.09	0.85–0.95
Number of cigarettes smoked per day			14.73 ± 9.49	12.96–16.44			18.47 ± 13.90	11.66–25.34
Years of non-smoking	6.18 ± 6.41	5.48–6.92			14.10 ± 9.91	10.04–18.16		

Notes: RNS: Referent non-smokers; RS: Referent smokers; NSHP: Non-smokers with hypertension; SHP: Smokers with hypertension.

**Table 2:** Prevalence of diseases in the study population.

Parameters		RNS		RS		NSHP		SHP		Total		Total	
		n	%	n	%	n	%	n	%	n	%	n	%
Diabetes	No	139	54.7	78	30.7	21	8.3	15	5.9	253	99.6	254	100
	Yes	0		1	1.3	0		0		1	0.4		
Diabetes treatment	No	139	54.7	79	31.1	21	8.3	15	5.9	254	100	254	100
	yes	0		0		0		0	5.9	0			
Hypertriglyceridemia	No	77	30.3	32	12.6	11	4.3	6	2.4	126	49.6	253	99.6
	Yes	62	24.4	47	18.5	9	3.5	9	3.5	127	50.0		
Hypercholesterolemia	No	120	47.2	69	27.2	16	6.3	12	4.7	217	85.4	254	100
	Yes	19	7.5	10	3.9	5	2.0	3	1.2	37	14.6		
Cholesterol treatment	No	139	54.7	79	31.1	20	7.9	15	5.9	253	99.6	254	100
	Yes	0		0		1	0.4	0		1	0.4		
Metabolic syndrome	No	127	50.0	67	26.4	14	5.5	10	3.9	218	85.8	254	100
	Yes	12	4.7	12	4.7	7	2.8	5	2.0	36	14.2		
Number of components of metabolic syndrome	0	54	21.3	26	10.2	0		1	0.4	81	31.9	254	100
	1	45	17.7	25	9.8	7	2.8	5	2.0	82	32.3		
	2	28	11.0	17	6.7	7	2.8	4	1.6	56	22.0		
	3	8	3.1	8	3.1	3	1.2	4	1.6	23	9.1		
	4	4	1.6	3	1.2	4	1.6	1	0.4	12	4.7		
Sintron treatment	No	139	54.7	79	31.1	21	8.3	15	5.9	254	100	254	100
	Yes	0		0		0		0		0			

Notes: RNS: Referent non-smokers; RS: Referent smokers; NSHP: Non-smokers with hypertension; SHP: Smokers with hypertension.

**Table 3:** Levels of zinc in serum, urine and Zn serum/urine quotients and oxidative stress markers in the study population.

Biomarkers ^(1)^	Non-hypertensive (n = 218)					Hypertensive (n = 36)					*p*-value ^(2)^	*p*-value ^(3)^
	Non-smoker (n = 139)		Smoker (n = 79)		*p* value	Non-smoker (n = 21)		Smoker (n = 15)		*p* value		
	Mean ± SD	95%CI	Mean ± SD	95%CI		Mean ± SD	95%CI	Mean ± SD	95%CI			
Zn serum (μmol/L)	13.39 ± 4.35	12.92–13.88	12.80 ± 3.77	12.10–13.50	0.314	12.32 ± 3.19	10.99–13.61	12.23 ± 4.30	10.08–14.32	0.942	0.281	0.601
Zn urine (μmol/L)	2.55 ± 2.91	2.27–2.93	2.59 ± 2.74	2.10–3.10	0.921	2.78 ± 2.13	1.94–3.66	2.69 ± 3.57	0.93–4.47	0.771	0.728	0.902
Zn serum/urine	43.74 ± 67.19	36.17–51.23	43.13 ± 69.24	30.40–55.79	0.949	28.79 ± 60.36	4.01–53.59	15.40 ± 17.36	6.84–23.96	0.336	0.337	0.082
8-OHdG	3.24 ± 1.72	3.05–3.43	3.34 ± 1.41	3.08–3.60	0.660	2.66 ± 0.79	2.34–2.98	2.03 ± 0.90	1.59–2.47	0.032	0.115	0.0008
MDA	0.63 ± 0.66	0.56–0.70	0.59 ± 0.70	0.46–0.72	0.674	0.62 ± 0.58	0.38–0.86	0.49 ± 0.22	0.38–0.60	0.342	0.948	0.314
GSH	16.30 ± 7.89	15.42–17.18	17.68 ± 7.52	16.30–19.06	0.208	16.28 ± 7.14	13.35–19.21	16.45 ± 6.18	13.41–19.49	0.941	0.991	0.553
GSSG	0.77 ± 0.71	0.69–0.85	0.75 ± 0.51	0.66–0.84	0.826	0.97 ± 0.79	0.65–1.29	0.80 ± 0.31	0.65–0.95	0.353	0.237	0.482
GSSG/GSH ^(4)^	15.67 ± 35.56	11.68–19.66	10.60 ± 24.49	6.11–15.09	0.262	21.44 ± 56.73	−1.85–44.73	10.81 ± 21.92	0.03–21.59	0.385	0.508	0.975
G Total	17.85 ± 8.20	16.93–18.77	19.18 ± 7.65	17.78–20.58	0.239	18.22 ± 7.22	15.26–21.18	18.05 ± 6.25	14.98–21.12	0.942	0.845	0.592

Notes: **^(1)^** Units: nmol/mmol creatinine; **^(2)^** *p*-value from the group of non-hypertensive non-smokers and the group of hypertensive non-smokers; **^(3)^** *p*-value from the group of non-hypertensive smokers and the group of hypertensive smokers; **^(4)^** Percentage of GSH oxidized.

The highest levels of the 8-OHdG marker were obtained for RS (3.34 ± 1.41 nmol/mmol creatinine), and the lowest levels went to SHP (2.03 ± 0.90 nmol/mmol creatinine). Statistically significant differences (*p* < 0.05) for the 8-OHdG marker were found between NSHP and SHP (*p* = 0.032) and between RS and SHP (*p* = 0.008). The highest value for the MDA marker was for RNS (0.63 ± 0.66 nmol/mmol creatinine), while the lowest value went to SHP (0.49 ± 0.22 nmol/mmol creatinine).

The highest GSH values were found in RS (17.68 ± 7.52 nmoles/mmoles creatinine) and the smallest values were obtained in NSHP (16.28 ± 7.14 nmoles/mmoles creatinine). NSHP obtained the highest GSSG values (0.97 ± 0.79 nmol/mmol creatinine) and the lowest ones were observed in the RS group (0.75 ± 0.51 nmoles/mmoles creatinine).

The highest GSSG/GSH quotient values were found in NSHP, 21.44 ± 56.73 (21.44% of the reduced glutathione in this group had been oxidized). The lowest values were obtained in RS, 10.60 ± 24.49 (only 10.60% of the glutathione in this group had been oxidized). RS presented the highest values of total glutathione (19.18 ± 7.65 nmol/mmol creatinine) and RNS showed the lowest values for this OS marker (17.85 ± 8.20 nmol/mmol creatinine).

### 3.2. Association of OS Markers with Zn Serum/Urine Quotient and Cotinine Levels

Table 4 shows the correlations between the OS markers with the Zn serum/urine quotient and with the cotinine level for the different study groups. Statistically significant correlations were found for the SHP group: a negative correlation was found between GSH and the Zn serum/urine quotient, with a value of *p* = 0.026. A positive correlation was observed between GSSG/GSH and the Zn serum/urine quotient, with a value of *p* = 0.010. A negative correlation was obtained between the total glutathione and the Zn serum/urine quotient, with a value of *p* = 0.020

### 3.3. OS Risk Factors

Table 5 shows an evaluation of risk of appearing values over the 75 percentile (P 75) for the OS markers in the study groups in relation to tobacco exposure and hypertension. Tobacco exposure increased the risk of high 8-OHdG values: OR = 1.27 (95%CI = 0.68–2.36) and AOR = 1.41 (95%CI = 0.73–2.73), when adjusted for different factors like age, gender, BMI, level of vitamin B12 intake and Zn serum/urine quotient.

The risk of values appearing over the 75 percentile for the OS markers in relation to hypertension increased for GSSG; OR = 2.80 (95%CI = 1.09–7.18) and for AOR = 3.06 (95%CI = 0.96–9.71), and both were statistically significant. Finally, the combined effect of tobacco exposure and hypertension increased the risk of obtaining high GSSG values: OR = 1.12 (95%CI = 0.34–3.76) and AOR = 1.73 (95%CI = 0.45–6.67).

**Table 4:** Correlation between oxidative stress markers with zinc serum/urine quotients and cotinine levels.

**Biomarkers**	**Zn Serum/Urine**							
	**RNS**		**RS**		**NSHP**		**SHP**	
	**C. Pearson**	***p*-value**	**C. Pearson**	***p*-value**	**C. Pearson**	***p*-value**	**C. Pearson**	***p*-value**
8-hydroxydeoxyguanosine (8-OHdG)	0.123	0.150	0.178	0.118	0.417	0.060	−0.217	0.436
Malondialdehyde (MDA)	0.049	0.565	−0.038	0.739	−0.184	0.424	−0.097	0.731
Reduced glutathione (GSH)	−0.067	0.434	0.166	0.146	0.156	0.500	−0.571	0.026
Oxidized glutathione (GSSG)	−0.087	0.310	−0.106	0.356	−0.245	0.285	−0.264	0.341
Percentage oxidized glut. (GSSG/GSH)	0.062	0.467	−0.145	0.205	−0.146	0.526	0.639	0.010
Total glutathione	−0.079	0.353	0.149	0.192	0.100	0.665	−0.591	0.020
**Biomarkers**	**Cotinine**							
	**RNS**		**RS**		**NSHP**		**SHP**	
	**C. Pearson**	***p*-value**	**C. Pearson**	***p*-value**	**C. Pearson**	***p*-value**	**C. Pearson**	***p*-value**
8-hydroxydeoxyguanosine (8-OHdG)	---	---	0.019	0.869	---	---	0.087	0.759
Malondialdehyde (MDA)	---	---	0.166	0.143	---	---	0.008	0.978
Reduced glutathione (GSH)	---	---	−0.045	0.696	---	---	−0.154	0.584
Oxidized glutathione (GSSG)	---	---	−0.084	0.461	---	---	0.000	1.0
Percentage oxidized glut. GSSG/GSH	---	---	0.044	0.700	---	---	−0.097	0.732
Total glutathione	---	---	−0.055	0.630	---	---	−0.152	0.589

Notes: RNS: Referent non-smokers; RS: Referent smokers; NSHP: Non-smokers with hypertension; SHP: Smokers with hypertension.

**Table 5:** Assessing the risk (OR) of oxidative stress markers in the non-hypertensive non-smoker population, in relation to: smoking, hypertension, and the combined effect of smoking and hypertension.

Biomarkers	P 75 (nmol/mmol creat.)	Non-hypertensive and Non-smoker/Non-hypertensive and Smoker				Non-hypertensive and Non-smoker/Hypertensive and Non-smoker				Non-hypertensive and Non-smoker/Hypertensive and Smoker			
		OR	95%CI	AOR ^(1)^	95%CI	OR	95%CI	AOR ^(1)^	95%CI	OR	95%CI	AOR ^(1)^	95%CI
8-OHdG	P < 4.10	1	---	1	---	1	---	1	---	1	---	1	---
	P > 4.10	1.27	0.68–2.36	1.41	0.73–2.73	0.15	0.02–1.19	0.16	0.02–1.34	---	---	---	---
MDA	P < 0.75	1	---	1	---	1	---	1	---	1	---	1	---
	P > 0.75	1.12	0.59–2.10	1.05	0.54–2.04	0.72	0.23–2.31	0.49	0.12–1.96	0.22	0.03–1.74	0.17	0.02–1.68
GSH	P < 21.34	1	---	1	---	1	---	1	---	1	---	1	---
	P > 21.34	0.81	0.43–1.51	0.86	0.44–1.68	0.78	0.28–2.16	0.77	0.24–2.49	0.62	0.19–1.95	0.47	0.13–1.69
GSSG	P < 0.87	1	---	1	---	1	---	1	---	1	---	1	---
	P > 0.87	0.98	0.51–1.86	0.98	0.50–1.91	2.80	1.09–7.18	3.06	0.96–9.71	1.12	0.34–3.76	1.73	0.45–6.67
GSSG/GSH	P < 6.7	1	---	1	---	1	---	1	---	1	---	1	---
	P > 6.7	0.72	0.36–1.43	0.64	0.32–1.30	1.54	0.58–4.14	1.92	0.59–6.27	0.77	0.20–2.89	1.25	0.29–5.38
Total glutathione	P < 23.14	1	---	1	---	1	---	1	---	1	---	1	---
	P > 23.14	1.09	0.57–2.06	1.02	0.51–2.02	1.60	0.60–4.31	1.57	0.49–4.99	1.17	0.35–3.91	1.58	0.41–6.19

Note: **^(^^1)^** Adjusted for gender, age, BMI, vitamin B12 intake and Zn serum/urine quotients. P75: 75 percentile.

## 4. Discussion

This study identifies that, despite having Zn intake levels that fell within the RDI for the Spanish population, low levels of zinc are associated with increased OS, and a role of zinc in cellular aging has been identified. It has also been observed that increased OS incurs a risk of 3.06 of developing high blood pressure after controlling for smoking, age, gender, BMI, level of vitamin B12 intake and Zn serum/urine quotient.

### 4.1. Hypertension and the Zinc Serum/Urine Quotient

Table 3 provides the Zn serum/urine quotient values which, as the literature indicates [10,23] can be used as an indication of hypertension. This pathological situation, in combination with the smoking habit, is associated with high zinc levels in urine and lower levels in serum. Indeed the Zn serum/urine quotient value lowered in the hypertensive population.

### 4.2. Hypertension, Smoking, Zinc Alterations and OS

The correlations observed in Table 4 can be interpreted together as a physiological regulation of the effects caused by oxidative stress due to hypertension. The negative correlation between GT and the Zn serum/urine quotient can be seen as an increase in glutathione production in response the Zn serum/urine quotient, which decreased due to increased hypertension mediated by OS. Glutathione is produced in the form of GSH. The antioxidant activity of GSH raises the GSSG levels and allows a partial recovery of the Zn serum/urine quotient, as reflected in the positive correlation between GSSG/GSH and the Zn serum/urine quotient.

The results obtained for GSH and GSSG offer us an idea of their inversely proportional character between groups and also inform about the higher OS in hypertensive subjects. The literature includes several studies that have analyzed variation in glutathione levels. In one of them [24], the authors found a larger quantity of GSH in the normotensive group than in the hypertensive group. The difference between these quantities was statistically significant. In our study, we observed that the highest GSH value was obtained for the non-hypertensive group, while the lowest value for this marker was obtained for the hypertensive group. Although these results were not significant, they fell in line with the results reported in former studies [24].

Another study [24] found correlations between hypertension levels and the peroxidase glutathione enzyme (GPx) and MDA levels. In our study, we found significant correlations for glutathione, which agree with the literature [25]. A former study [26], which considered that obesity indicates hypertension, observed differences between the levels of several OS markers between obese population groups and a normoweight group.

Table 4 presents the correlation between OS markers with cotinine levels. No statistically significant results were obtained for the smokers groups. This could be due to the fact that while OS implies permanent damage, cotinine values vary over time despite it being the most suitable way to analyze tobacco smoking since presence of cotinine in the organism lasts much longer than nicotine. Some studies have analyzed the relationship between OS and smoking habit. One of them [27] obtained the lowest GSH and the highest GSSG values in a group of smoking women compared with a control group. These results indicate increased OS in relation to smoking, which agrees with the results obtained in our work.

Another study [28] analyzed the OS marker 8-OHdG level in a population of administration workers, classified according to their smoking habit. Smokers presented the highest levels, non-smokers obtained the lowest values and ex-smokers presented intermediate levels. The same study [28] obtained an increased risk for 8-OHdG appearing when adjusting for CO concentration. These results agree with our study, where we found an increased risk of some OS markers (Table 5).

Previous studies [18] have been found that the hypertensive population had higher levels of OS markers, as well as a larger amount of zinc in serum. Our study obtained similar results to another study [29] as we found a correlation between decreased levels of zinc in serum and increased levels in some OS markers (Table 4).

Others studies have analyzed the combined effect of smoking and occupational exposure to metals on the level of OS markers [29,30] and saw that OS markers obtained higher values for workers exposed to metals, regardless of them being smokers or non-smokers. These results possibly indicate that the oxidative effect of tobacco is masked due to occupational exposure to metals. One of them [29] also studied variation in serum zinc levels according to occupational exposure and smoking, and obtained the highest values in smokers exposed to metals. In our study, this was a controlled confounding factor because we worked with a large database that covered populations of different ages and numerous occupations.

Other than the aforementioned processes during which OS appeared, there are other less known ones that concern the toxicity of zinc nanoparticles. Most technologies have enabled advances to be recently made in the nanoscience field. Zinc, in the form of zinc oxide (ZnO) nanoparticles, can be incorporated into the organism in several ways. In this form it can contribute to OS, as former studies have previously indicated [31]. Among the factors that determine cytotoxicity caused by ZnO nanoparticles, the dimensions of particles [32], the dose in which they have been incorporated [33] or the form they take [33] have been found. As OS inducers, ZnO nanoparticles can harm DNA, mitochondria and cells membranes [34], like the other aforementioned markers. During the ZnO-mediated cytotoxicity regulation process, suppressor gene p53 intervenes [35,36] which can activate antioxidant genes or cell apoptosis in accordance with the OS level.

### 4.3. Study Limitations

There are some limitations in the present study. First, the sample size with whom this work was finally conducted (254 individuals) was relatively small despite the large initial sample size (1500 people) as a result of applying rigorous inclusion criteria. Nonetheless, this makes the obtained results more reliable because a series of confounding factors was controlled. Second, our study did not consider the exsmoker condition as a study group as other studies have done [28] because we worked only with smokers and non-smokers categories [20]. The reason why we did not consider exsmokers was because of their high heterogeneity as they presented various past smoking habit intensities and different times since they had stopped smoking, which would have make it a group with biased information that would be difficult to interpret. Finally, although we observed a correlation between alteration in physiological zinc levels and damage caused by OS, future studies are required to prove this relationship.

## 5. Conclusions

The present work identifies a drop in zinc levels in serum and an increase in urine zinc levels. These variations in physiological zinc levels correlate with increased OS and blood pressure. The literature [37] indicates that regulation of zinc levels can be a way of improving the organism’s physiology.
